# Adoptive cellular immunotherapy for refractory childhood cancers: a single center experience

**DOI:** 10.18632/oncotarget.27242

**Published:** 2019-10-22

**Authors:** Michael Merker, Michael Torsten Meister, Annekathrin Heinze, Andrea Jarisch, Jan Sörensen, Sabine Huenecke, Melanie Bremm, Claudia Cappel, Thomas Klingebiel, Peter Bader, Eva Rettinger

**Affiliations:** ^1^ Division of Stem Cell Transplantation and Immunology, Department of Children and Adolescent Medicine, University Hospital Frankfurt, JW Goethe University, Frankfurt am Main, Germany; ^2^ Princess Máxima Center for Pediatric Oncology, Utrecht, Netherlands

**Keywords:** adoptive cellular immunotherapy, allogeneic, solid tumors, childhood, HSCT

## Abstract

Prognosis of refractory childhood cancers despite multimodal treatment strategies remains poor. Here, we report a single center experience encountered in 18 patients with refractory solid malignancies treated with adoptive cellular immunotherapy (ACI) from haploidentical or matched donors following hematopoietic stem cell transplantation. While seven patients were in partial and six in complete remission (CR), five patients suffered from relapsed diseases at the time of ACI. 1.5-year probabilities of overall survival (OS) and progression-free survival (PFS) were 19.5% and 16.1% for all patients. Patients in CR showed estimated 1.5-year OS and PFS of 50.1% and 42.7%, respectively. CR was induced or rather sustained in ten children, with two still being alive 9.6 and 9.3 years after ACI. Naïve, central and effector memory T-cells correlated with responses. However, the majority of patients relapsed. Cumulative incidence of relapse was 79.8% at 1.5 years. Acute graft versus host disease (aGVHD) occurred in nine of 18 patients (50%) with aGVHD grade I–II observed in six (33%) and aGVHD grade III seen in three (17%) patients, manageable in all cases.

Altogether, study results indicate that donor-derived ACI at its current state offers palliation but no clear curative benefit for refractory childhood cancers and warrants further improvement.

## INTRODUCTION

Prognosis of pediatric patients with intermediate- or even high-risk sarcomas such as rhabdomyosarcoma (RMS) synovial sarcoma (SS), or Ewing sarcoma (ES) as well as with neuroblastoma (NB), hepatoblastoma (HBL) or nasopharyngeal carcinoma (NPC) has improved dramatically over the last decades by first line treatments [1–5].

However, adolescents and young adults with metastatic alveolar subtype RMS including bone or bone marrow involvement still are incurable [6]. Likewise, prognosis for patients with primary metastatic SS (North American Intergroup Rhabdomyosarcoma Study Group, IRS group IV tumor) remains dismal [7], and the overall survival (OS) rate of ES patients diagnosed with primary metastases is below 30% as well [3].

The International Neuroblastoma Risk Group (INRG) defined 16 different patient risk categories based on the INRG stage, tumor histology, and grade of tumor differentiation, MYCN status, 11q status, ploidy, and anaplastic lymphoma kinase amplification [8]. More than half of high-risk patients (International *Neuroblastoma Staging System* (INSS) stage 4 and INRG stage M patients *≥*18 months of age and all NB-patients with a MYCN amplification) die from disease despite intensive multimodal treatment, including chemotherapy, surgery and myeloablative chemotherapy with autologous stem cell rescue, as well as 131-metaiodobenzylguanidine therapy or external beam radiation. The combination of the ch14.18/SP2/0 anti-GD2 antibody (Dinutuximab), CSF2 (colony stimulating factor 2), interleukin (IL)-2 and isotretinoin may be provided in case of refractory disease [9].

HBL patients who present with metastases as well as older patients (≥8 years old at diagnosis) and patients with low alpha-fetoprotein levels (<100 ng/mL) have also unfavorable outcomes. Pre- and post-op chemotherapy, resection of metastases, liver transplantation, or transaterial chemoembolization has been effective in some very high risk HBL patients. However, conventional treatments are not successful in case of disease recurrence [10]. Altogether, subgroups of solid tumor patients still face poor prognosis [3, 6, 7]. Therefore, novel therapeutic strategies are required for these patients.

Immune checkpoint inhibitors have not yet shown efficacy in refractory pediatric solid tumors likely due to low mutational burden and the tumor microenvironment involved in acquired resistance of tumors. Although not yet a standard of care, targeted cancer therapies that interfere with specific molecules involved in *cancer* growth, spread and survival may represent the next generation of cancer treatment. Hence, panel sequencing of drug-able molecular alterations and gene expression profiling are or will be assessed in current or upcoming clinical trials. However, the lack of ideal targets or the fact, that drugs are not yet approved for clinical use in childhood tumors are limiting this strategy.

Replacing the immune system by an allogeneic hematopoietic stem cell transplantation (HSCT) performed on a compassionate use basis in refractory solid malignancies at many pediatric transplant centers has been proposed as a potentially curative therapy due to its presumable graft versus tumor (GVT) effect [11] in patients with metastatic and relapsed ES [12], NB [11, 13, 14], and HBL [15], accompanied with moderate treatment-related toxicity.

Based on these promising data, we additionally performed consecutive donor-derived ACI in allogeneic HSCT-patients with refractory or relapsed solid malignancy to further increase anti-tumor efficacy after transplantation. ACIs comprised of donor lymphocyte infusions (DLI), natural killer (NK) cell [16] or cytokine-induced killer (CIK) cell infusions [17] generated from the original stem cell donors. Here we present safety and efficacy data as well as immune monitoring data and outcome of allogeneic HSCT-recipients undergoing donor-derived ACI.

## RESULTS

### Patient characteristics

Between October 1st, 2003 and January 1st, 2014, a total of 18 patients were enrolled in this single center prospective study, conducted in Frankfurt/Main, Germany. Eight patients with RMS, one patient with SS, two patients with ES, five patients with NB, one patient with HBL, and one patient with NPC were enrolled (Table 1). The median age at diagnosis was 11.8 years (range, 1.8 – 25.1 years) and the median time from diagnosis to transplantation 20.0 months (range, 6.5 – 78.3 months). Hence, median age at allogeneic HSCT was 13.2 years (range, 3.2 – 27.2 years). Of note, patient no. 16 developed a secondary acute myeloid leukemia (AML) and received an allogeneic HSCT for secondary AML 21 months after being diagnosed with ES. This patient relapsed 46 months after the primary ES diagnosis and received donor-derived ACI for relapsed ES a long time (1123 days) after allogeneic HSCT (Supplementary Table 1). More than one third of the remaining patients enrolled in this study had achieved complete remission (CR) before HSCT (7 of 17, 41%), while another seven of 17 (41%) patients had obtained at least very good partial or partial response (VGPR or PR), and three patients (18%) suffered from relapsed or refractory diseases at the time of transplantation.

**Table 1:** Patient characteristics, *n* = 18

**Gender**	
female	4
male	14
**Median age, y (range)**	
at diagnosis	11.8 (1.8–25.1)
at allogeneic HSCT	13.2 (3.2–27.2)
**Median time to transplantation, m (range)**	
from diagnosis	20.0 (6.5–78.3)
**Disease, n**	
Rhabdomyosarcoma	8
Ewing sarcoma	2
Synovial sarcoma	1
Neuroblastoma	5
Hepatoblastoma	1
Nasopharynx carcinoma	1
**Disease status before transplantation, n**	
CR1	3
CR2	3
CR>2	1
VGPR	1
PR	6
rlps	4
**Donor, n**	
MF/UD	2
MMFD	16
**Conditioning regimen, n**	
flu/thio/mel + OKT3	13
flu/thio/mel + ATG	2
clo/eto/cyc + flu/thio/mel + campath	2
n. a.	1
**Median follow-up after ACI, m (range)**	8.5 (1.5–115.1)
**Best response to ACI, n**	
CR	8
SD	9
rlps	1

Abbreviations: HSCT, Hematopoietic stem cell transplantation; CR, complete remission; VGPR, very good partial remission; PR, partial remission; SD, stable disease; rlps, relapse; MF/UD, matched family/unrelated donor; MMFD, mismatched family donor; flu, fludarabine; thio, thiotepa; mel, melphalan; clo, clofarabine; eto, etoposidem; cyc, cyclophosphamide; y, year; m, month; ACI, adoptive cellular immunotherapy.

After long lasting consultation, it was considered problematic to use volunteer unrelated donors for such an experimental approach not knowing whether patients might benefit from allogeneic HSCT at all. Therefore, family donors, parents and adult siblings, were allowed to be donors for these patients. Sixteen of 18 (89%) cases were grafted from haploidentical donors with 5 of 10 human leukocyte antigen (HLA)-mismatches, whereas the remaining two cases (11%) had matched family or matched unrelated donors (Table 1).

Thirteen of 18 (72%) cases received uniform conditioning consisting of fludarabine, thiotepa, melphalan, and muromonab-CD3 (OKT-3^®^). After OKT-3® was no longer available in January 2011, conditioning regimen had to be switched to fludarabine, thiotepa, melphalan, and anti-thymocyte globulin (ATG) in two of 18 (11%) patients. Another two (11%) patients enrolled in this study received a conditioning regimen consisting of clofarabine, etoposide, and cyclophosphamide, followed by fludarabine, thiotepa, melphalan plus alemtuzumab (Supplementary Table 1).

### ACI treatment and response

ACI started at a median of 54 days (range, 3–1123 days) after allogeneic HSCT. Of note, patient no. 16 had received allogeneic HSCT for secondary AML 1123 days before donor-derived ACI was given for relapse of ES. DLI was applied in five patients, NK cell treatment was offered to another six patients of whom four suffered from NB, while CIK cell therapy was given to seven patients. Most of the CIK cell treatments were applied during relapsed disease (5 of 7 cases, 85%), while DLI and NK cell treatment were given either during CR or PR. Median follow up (FU) after ACI was 8.5 months (range, 1.5 – 115.2 months). Best response to ACI was (sustained) CR in eight of 18 (44%) patients (DLI, 1 of 5 patients; NK, 2 of 6 patients; CIK cell infusions, 5 of 7 patients), and stable disease in nine of 18 (50%) cases (DLI, 4 of 5 patients; NK, 3 of 6 patients; CIK cell infusions, 2 of 7 patients), while only one patient with NK cell infusions (5.5%) showed no responses to ACI (Table 1 and 2 and Supplementary Table 1).

### Relapse and survival

**Figure 1:**
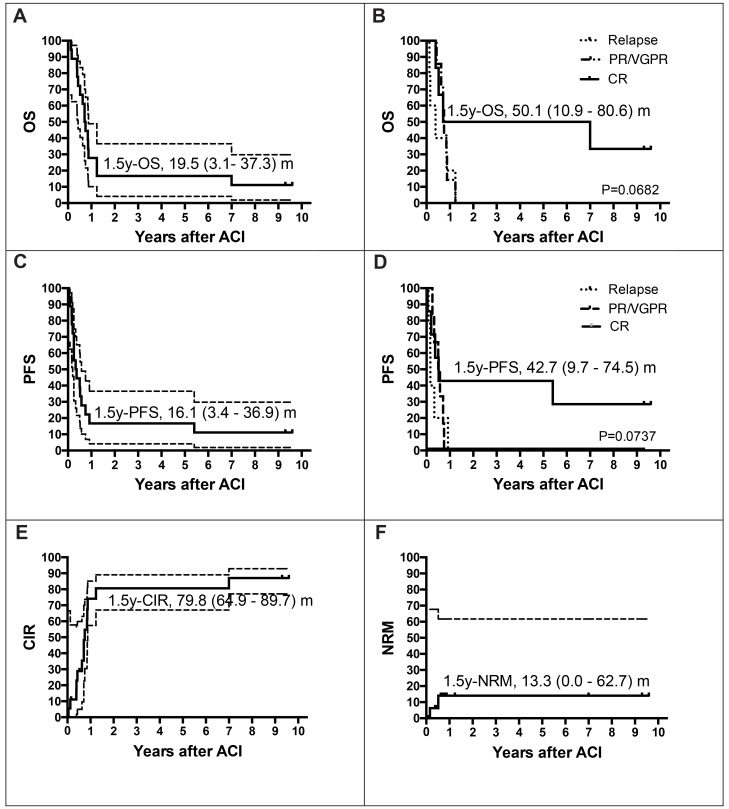
Estimates of overall survival rate (OS), event free survival (EFS), relapse and non-relapse mortality (NRM). Patients at risk, *n* **=** **18.** Probability of overall survival (pOS) (**A**) and progression-free survival (pPFS) (**C**) of all patients (*n =* 18) 1.5 years after adoptive cellular immunotherapy (ACI): pOS, events, *n =* 16; 0.16 (95% CI, 0.04–0.37); pPFS, events, *n =* 16; 0.16 (95% CI, 0.03–0.37). Probability of overall survival (pOS) (**B**) and progression-free survival (pPFS) (**D**) of patients in CR at the time of ACI (*n =* 6) 1.5 years after ACI: pOS, events, *n =* 4; 0.50 (95% CI, 0.11–0.81); pPFS, events, *n =* 5; 0.43 (95% CI, 0.1–0.75). (**E**) Cumulative incidence of relapse (CI-R, *n =* 18; events, *n =* 14; 0.80; 95% CI 0.65–0.90) and (**F**) NRM (CI-NRM, *n =* 18; events, *n =* 2; 0.13; 95% CI 0.0–0.63) is given at 1.5 years after ACI for all patients.

**Figure 2:**
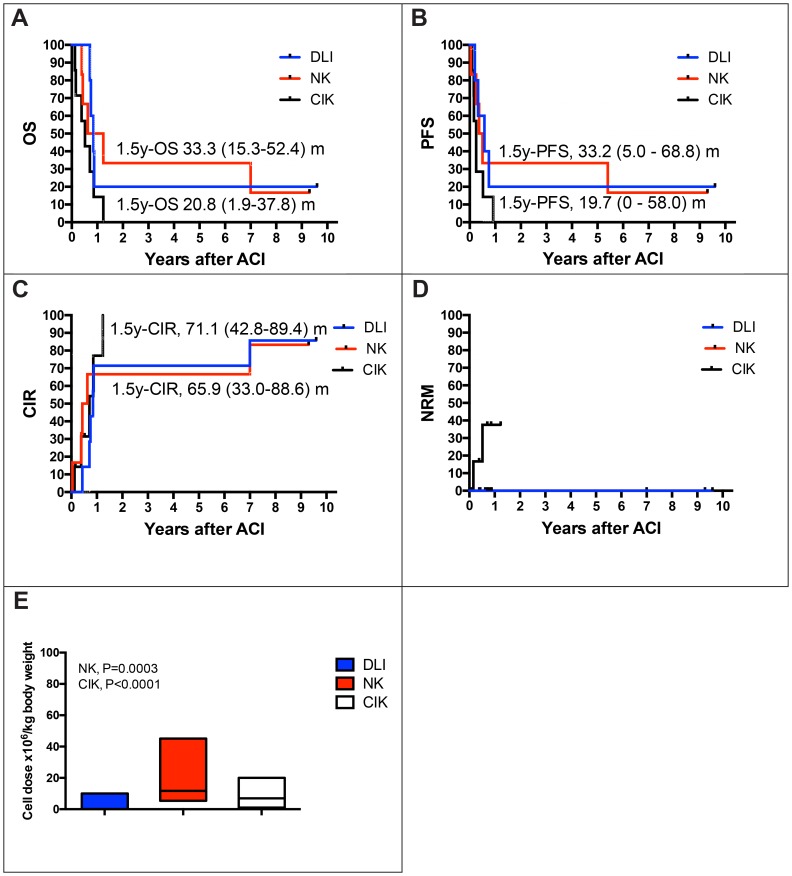
Estimates of overall survival rate (OS), event free survival (EFS), relapse and non-relapse mortality (NRM) with regard to type of ACI. Patients at risk, *n* **=** 18. Subanalysis of probability of overall survival (pOS) (**A**) and progression-free survival (pPFS) (**B**) of patients 1.5 years after cellular immunotherapy (DLI; NK, CIK cell therapy): pOS DLI, events, *n =* 4; 0.21 (95% CI, 0.02–0.38); pOS NK, events, *n =* 5; 0.33 (95% CI, 0.15–0.52); pPFS DLI, events, *n =* 4; 0.20 (95% CI, 0.0–0.58); pPFS NK, events, *n =* 5; 0.33 (95% CI, 0.05–0.69). (**C**) Cumulative incidence of relapse (CI-R, DLI events, *n =* 4; 0.71; 95% CI 0.43–0.89; CI-R, NK events, *n =* 5; 0.66; 95% CI 0.33–0.87) and (**D**) NRM occurred in 2 patients with CIK cell treatment but was not clearly related to immune cell infusions. (**E**) Immune cell doses applied by DLI, NK and CIK cell therapy shown as floating bars (min to max, with line at median). NK and CIK cell doses were significantly increased compared to DLI.

In summary, 1.5-year overall survival (OS) estimate was 19.5% and 1.5-year progression-free survival (PFS) estimate was 16.1% (Figure 1A and 1C). The vast majority of events observed in this study were relapses and disease progressions that appeared within the first five months after donor-derived ACI. Most of the patients succumbed shortly thereafter. Of note, two of the 18 patients enrolled in this study are still alive and in CR 9.3, and 9.6 years after ACI (one patient with SS and DLIs, and one patient with NB and NK cell therapy; Supplementary Table 1 and Table 2), respectively. Both patients were in CR at the time of allogeneic HSCT as well as at the time of ACI. Another patient with NB, who received his allogeneic HSCT in partial response to conventional treatment, responded to allogeneic HSCT and was offered cellular immunotherapy treatment with IL-2 stimulated NK cells in CR. Of note, this patient did not relapse until 5.8 years after ACI. CIK cell infusions from haploidentical donors induced CR in another five children with RMS, ES, and NPC. Three had active diseases, one patient was in a VGPR and another one in CR at the time of ACI. One patient with relapsed RMS and one patient with relapsed ES remained in CR for 11 and 2 months without any signs of acute graft versus host disease (aGVHD), respectively. One patient with NPC died due to respiratory failure in the context of pneumonia, and one with RMS due to multiple organ failure in the context of cumulative toxicity, and viral infection after successful immunosuppressive treatment of aGVHD grade III, both were still in remission. Furthermore, another patient with RMS and aGVHD grade III remained in remission as long as signs of aGVHD were present but relapsed after initiation of steroid treatment. Despite induction of CR by CIK cell infusions, this did not result in improved survival of respective heavily pretreated patients (Figure 2). Altogether, disease status at allogeneic HSCT did not influence outcome after ACI, but differences were observed by comparing the status of diseases at the time of ACI. Relapse, PR/VGPR, or CR at the time of ACI resulted in an median time to disease progression of 2.0 months (range, 1.0–11 months), 4.5 months (range, 0.5–9.0 months), and 37.9 months (range, 2.4–115.2 months) (*P* = 0.0136), while patients were followed by a median of 4.6 months (range, 1.5–14.8 months), 8.0 months (range, 0.5-10.2 months), and 46.3 months (range, 4.7-115.2 months), respectively (*P* = 0.0051). Hence, patients in CR at the time of ACI showed 1.5y-OS and 1.5y-PFS probabilities of 50.1 and 42.7% (Figure 1B and Figure 1D), patients with relapse, PR or VGPR at the time of ACI progressed within 0.5 to 11 months and finally died two to 15 months after first ACI. In summary, probability of cumulative incidence of relapse (CIR) was 79.8% at 1.5 years after ACI (Figure 1E).

### Immune cell dose, infusion-related toxicity and GVHD

Immune cell doses applied by DLIs ranged from 0.025 × 10^6^ to 0.29 × 10^6^/kg in the mismatched and from 1.0 × 10^6^ to 10.0 × 10^6^/kg T cells in the matched donor setting, respectively (Supplementary Table 1 and Table 2). Applied NK and CIK cell doses were significantly increased (NK, *P* = 0.0003; CIK, *P* < 0.0001) and ranged from 1.3 × 10^6^ to 45.1 × 10^6^/kg NK cells and from 1.0 × 10^6^ to 19.3 × 10^6^/kg T cells among CIK cells, respectively (Figure 2E). Cumulative doses of immune cells were 2.4 ± 8.8 × 10^6^/kg, 39.6 ± 29.9 × 10^6^/kg, and 19.9 ± 16.8 × 10^6^/kg applied with a median number of 2.33, 2.60 or 2.29 DLI, NK or CIK cell infusions, respectively.

All infusions were well tolerated, and no acute toxicities were seen (Table 2). In the FU period, aGVHD occurred in nine of 18 patients (50%) with aGVHD grade I-II observed in six (33%) and aGvHD grade III seen in three (17%) patients (Table 3). Grade I-III aGVHD was seen in one patient with DLI, in five patients with NK and in three patients with CIK cell treatment. Grade I aGVHD was also observed in one CIK cell treated patient, while another two patients developed grade III aGVHD. Of note, aGVHD was manageable in all patients. Treatment of aGVHD including mycophenolatemofetil (MMF) or cyclosporine A (CsA). Patients with aGVHD grade III also received steroids and in two cases multiple administrations of mesenchymal stromal cells (MSC), while one of these two patients furthermore received extra corporal photopheresis (ECP) as GVHD-therapy.

**Table 2:** ACI and outcome, *n* = 18

ACI	DLI	NK	CIK
Number of pts.	*n =* 5 (28%)	*n =* 6 (33)	*n =* 7 (39%)
Cumulative dose of immune cells ×10^6^/kg	2.41 ± 4.80	39.64 ± 29.93	19.92 ± 16.77
Median number of infusions/pts.	2.33	2.60	2.29
aGVHD			
Grade I-II	*n =* 1 (5.5%)	*n* = 4 (22%)	*n* = 1 (5.5%)
Grade III	none	*n =* 1 (6%)	*n =* 2 (11%)
GVHD therapy	CsA, MMF	CsA, MMF, Steroids,	CsA, MMF, Steroids, MSC, ECP
Best response			
CR	1 (5.5%)	2 (11%)	5 (28%)
SD	4 (22%)	3 (17%)	2 (11%)
Rlps/progression		1 (5.5%)	
Last FU			
CR	1 (5.5%)	1 (5.5%)	
Rlps	4 (22%)	5 (28%)	5 (28%)
NRM			2 (11%)

Abbreviations: CR, complete remission; SD, stable disease; rlps, relapse; NRM, non-relapse mortality; ACI, cellular immunotherapy; NK, natural killer cells; DLI, donor lymphocyte infusion; CIK cells, cytokine-induced killer cells; FU, follow up; GVHD, graft vs. host disease; CsA, cylosporine A; MMF, mycophenolatmofetil; MSC, mesenchymal stroma cells; ECP, extra corporal photopheresis.

**Table 3:** Infusion-related toxicity

Total number of patients	*n* = 18 (100%)
**Acute toxicity**	none
**aGVHD**	
none	*n* = 9 (50%)
Grade I-II	*n* = 6 (33%)
Grade III	*n* = 3 (17%)
Grade IV	none
**cGVHD**	
limited	*n* = 1 (5%)
**Infusion-related deaths**	No clear infusion-related deaths

Abbreviations: GVHD, graft vs. host disease; aGVHD, acute graft vs. host disease; cGVHD, chronic graft vs. host disease.

### Non-relapse mortality

The overall observed non-relapse mortality (NRM)-rate was 11% (2 of 18 patients) with a 1.5y-predicted rate of 13.3% (Figure 1F). One patient died due to respiratory failure in the context of pneumonia, and one due to multiple organ failure in the context of cumulative toxicity, and viral infection after successful immunosuppressive treatment of aGVHD. Hence, NRM in both cases was not clearly due to ACIs.

### Immune reconstitution monitoring

**Figure 3:**
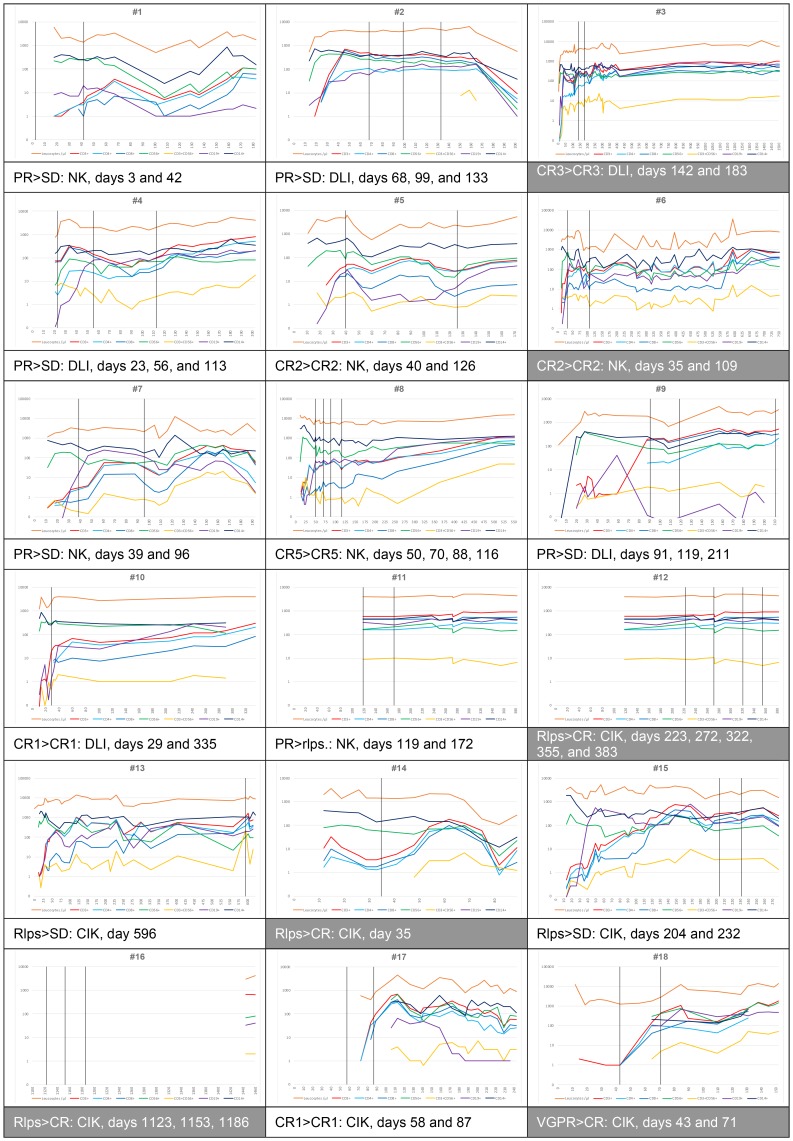
Immune recovery after allogeneic HSCT. Available data are shown. All patients were full donor chimerism. Immune recovery (IR) of leukocytes was rapid after allogeneic HSCT. Furthermore, occurrence of CD3^+^CD56^-^ (T cells), CD3^-^CD56^+^ (NK cells), and CD3^+^CD56^+^ (T-NK cells), CD19^+^ B cells, as well as CD14^+^ monocytes was monitored. Reconstitution of all cell types was increased or stabilized after IT (indicated by vertical lines) including helper (CD3^+^CD4^+^) and cytotoxic (CD3^+^CD8^+^) T cells. Immune cell reconstitution was increased when T cell numbers in the peripheral blood were below 1000/μl at the time of infusions (patient #1, #4, #6, #7, #8, #9, #10, #14, #17, and #18). Patients #3, #6, #8, #12, #14, #16, #17, and #18 were considered responders.

Overall, immune reconstitution (IR) was rapid with leukocytes recovery starting 11 days after allogeneic HSCT (Figure 3). Reconstitution of all cell types (T, NK, T-NK, and B cells as well as monocytes) was increased or stabilized after ACI including helper (T4) and cytotoxic (T8) T cells. Immune cell reconstitution was further increased by ACI, when T cell numbers in the peripheral blood were below 1000/μl at the time of infusions (patient # 1, 4, 6, 7, 8, 9, 10, 14, 17, and 18). However, improved immune cell engraftment-controlled diseases in most cases but was not correlated with improved PFS or OS.

**Figure 4:**
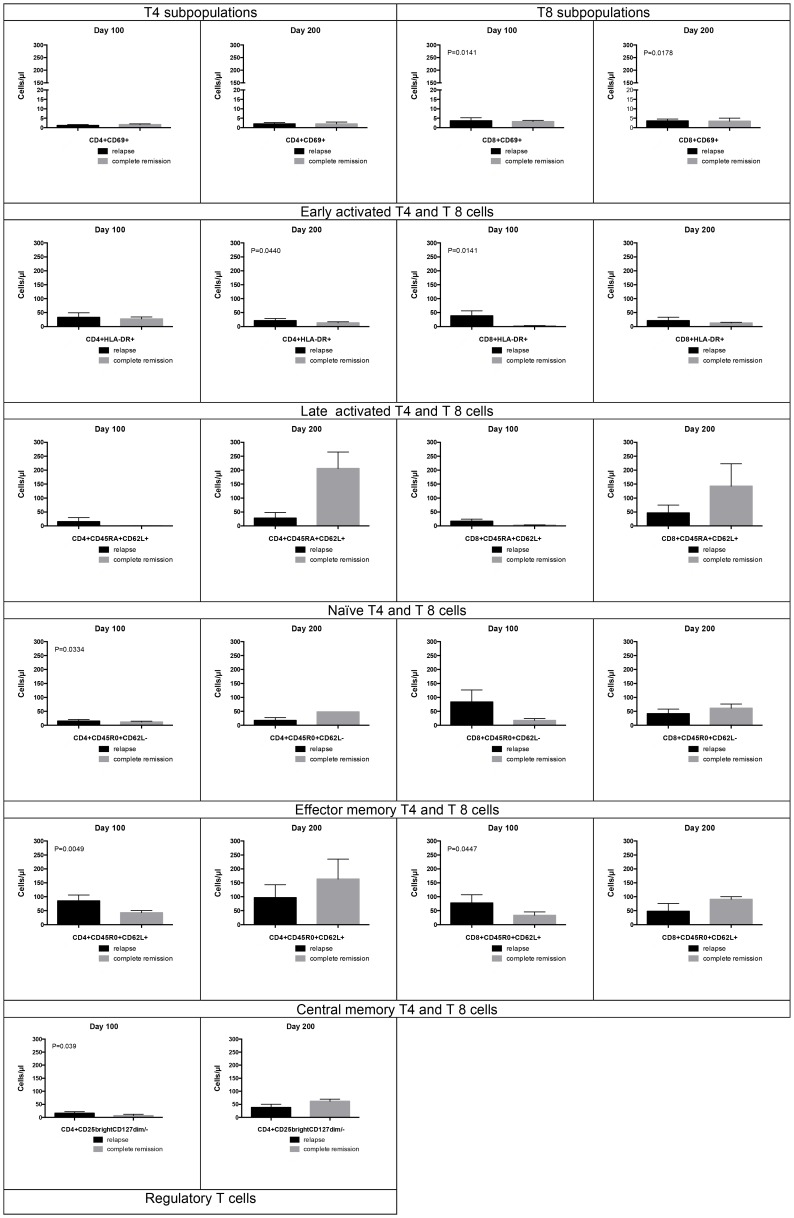
T cell effector subpopulations. This shows results of T cell effector subpopulations at 100 days and 200 days after transplant as bars (Mean with SEM). 5 of 18 patients hadn’t received ACIs by day 100 and, 6 of 18 patients received NK cells, without impacting T cell subpopulations. We observed lower numbers of regulatory T cells at day 100 (*P* = 0.039) and higher numbers of naïve, CM, and EM T4 and T8 cells in patients with effective and sustained immune responses 200 days after allogeneic HSCT compared to patients without responses, but differences were not statistically significant. Of note, early and late activated T4 and T8 cells were significantly increased in patients without sustained immune responses. Lower circulating immune populations in patients with immune responses may reflect clearance of the tumor and a contracted effector population whereas in those with relapse there might be a tumor-driven ‘inflammatory’ state.

Interestingly, we observed lower numbers of regulatory T cells at day 100 (*P* = 0.039) and higher numbers of naïve, central memory (CM), and effector memory (EM) T4 and T8 cells in patients with effective and sustained immune responses 200 days after allogeneic HSCT compared to patients without responses, but differences were not statistically significant. Of note, early and late activated T4 and T8 cells were significantly increased in patients without sustained immune responses (Figure 4).

## DISCUSSION

Despite prognosis for pediatric patients with solid tumors has improved substantially over the last years [1, 3] outcome of patients with relapsed or refractory diseases remains poor [3, 6, 7]. There is no clear and convincing evidence yet to support allogeneic HSCT, and therefore the role for donor-derived ACIs in children and adolescents suffering from these cancers. However, various preliminary clinical studies indicated a potential GVT effect in patients with metastatic or relapsed ES, NB, and HBL undergoing allogeneic HSCT, accompanied by moderate treatment-related toxicity. Potent antitumor responses were suggested to be provided by the newly established donor-derived immune system [18]. Based on these promising, preliminary reports, 18 patients with refractory or relapse childhood cancers in this study were offered allogeneic HSCT and in addition donor-derived ACIs to further increase GVT.

In our previous report of allogeneic HSCT in soft tissue sarcoma patients, event-free survival and OS at three years were 12% and 20%, respectively [19]. Outcome in most cases was limited by relapse. This might be due to the clinically aggressive nature of the analyzed diseases [1, 3, 6, 7]. Indeed, four of 18 patients in this actual study showed disease progression after HSCT, another two patients had improved, and the remaining 12 patients had stable diseases when starting ACI. Predicted 1.5y-PFS and 1.5y-OS were 19.5% and 16.1%, respectively. Of note, 1.5y-PFS and 1.5y-OS increased to 50.1% and 42.7% in patients that were in remission at the time of ACI. Although these patients certainly represent a heavily preselected group, these observations support a potential role of ACI for control of minimal residual disease states.

Conditioning and serotherapy which was altered for patients enrolled after January 2011, still might have been present by the time of ACI. Therefore, ACI in median was started 54 days after allogenic HSCT. IR of NK cells, T cells or T-NK cells was improved in patients who showed anti-tumor responses. This effect was even more pronounced in patients with low lymphocyte numbers at the time of ACI, indicating that persisting lymphopenia may have facilitated immune cell recovery in respective patients. Interestingly, patients with CR showed significant lower numbers of Tregs at day 100 compared with patients that experienced relapse. Furthermore, higher numbers of naïve, CM, and EM T4 and T8 cells were observed in patients with effective and sustained immune responses compared to patients without responses in whom early and late activated T4 and T8 cells were significantly increased. Lower circulating immune populations in patients with immune responses may reflect clearance of the tumor and a contracted effector population whereas in those with relapse there might have been a tumor-driven ‘inflammatory’ state. Therefore, delayed IR that might have resulted in temporary impaired immune surveillance of the tumor—despite or before ACI—could not completely explain the high rate of relapses observed in this study. Furthermore, high tumor burden, as well as the histopathological structure, the lack of immunogenicity, and the complex immunosuppressive microenvironment of solid tumors, capable of suppressing even strong immune responses may as well have limited anti-cancer cellular therapy.

Refractory tumors showing no or only low major histocompatibility complex (MHC) surface molecules combined with the expression of the NKG2D-target MICA, in principle can be treated by NK [20, 21] and CIK cells. However, existing clinical scale NK cell manufacturing protocols lack sufficient expansion rates, are time, personnel and cost extensive, impeding repetitive donor-derived NK cell therapy in the early post-transplant period. In contrast, clinical expansion of CIK cells with diverse T and NK cell receptor specificities [22] is robust and easy, and therefore once available at our center on August 11, 2011 CIK cell treatment was offered to all consecutive patients. The situation is certainly more clear in a similar paper by Schobrt *et al.* [23] as it focuses on ES and RMS only, treated only with DLI and is thus able to draw the conclusions, that HLA-mismatched DLIs were associated with a trend towards increased survival after allogeneic HSCT and increased post-relapse survival compared to HLA-matched DLI (23 versus 3 months). However, a subdivision of the current paper into the separate disease entities or the different types of ACIs to enable a clearer message due to the low number of patients and the diversity of tumors treated with different conditioning regimens and matched as well as mismatched donors from the statistical point of view was not reasonable.

Five of our patients received DLI, six patients were given NK cell infusions, and seven patients were offered CIK cell treatment. Interestingly, four of seven patients with relapse or VGRP treated with CIK cells achieved CR, which in one patient lasted up to 11 months clearly indicating the anti-tumor potential of CIK cells, while another two of six patients who received NK cells showed a noteworthy long-lasting response. One patient is alive 9.3 years after HSCT whereas the second patient remained in CR for 5.8 years. However, two of the only surviving patients in this study had received DLI and NK cell treatment while being in CR. Hence, the potential anti-tumor benefits in these patients may have been also due to the HSCT and not only due to the respective ACI.

All infusions were well tolerated, and no acute toxicities were seen, demonstrating that cell therapies are feasible and safe and can be given as an outpatient. Furthermore, study results confirmed a remarkably low incidence of GVHD, which was manageable in all cases. Yet, of the 18 patients three developed severe grade III aGVHD involving the gut. Two of these three patients had received CIK cell and another NK cell treatment, both associated with limited risk for GVHD induction. However, occurrence of grade III aGVHD may have been promoted by the haploidentical stem cell transplantation setting per se. Another six patients who received DLI, NK cell, and CIK cell treatment developed grade I-II aGVHD. Two of these are the only surviving patients in this study indicating that GVT was mediated by GVHD. Both patients with severe grade III aGvHD remained in CR until either immunosuppressive treatment was started (including steroids) or death due to NRM occurred. Hence, the potential anti-tumor benefits may have been mitigated by immunosuppressive drugs and not necessarily by lack of efficacy of the respective ACI. NRM was observed in another patient in CR treated with CIK cells who showed mild signs of aGVHD (grade I). However, due to the low number of patients and the heavy anti-tumor pretreatment combined with viral infection or reactivation, both influencing toxicity, no conclusion on safety and efficacy could be drawn from this study. Nevertheless, evidence was provided that CIK cell treatment could protect patients from relapse at the cost of increased toxicity when being combined with allogeneic HSCT.

In summary, the results of this study indicate that ACI in its current state offers palliation, but no clear curative benefit compared to established standard therapies [24]. However, there may be subsets of patients who may benefit from ACI, and biomarkers are sorely needed to identify these patients. We identified that CIK cells caused or sustained CR in five of seven and NK cells and DLI led to a response in two of six and one of five patients, respectively, suggesting repeated infusions early after HSCT with prudent dose escalation may be needed. Also, the use of steroids for GVHD may have unintentionally impaired IR and anti-tumor efficacy, suggesting steroid-sparing regimens are needed to complement ACI. Severe side effects of an allogeneic HSCT such as aGVHD further increased by allogeneic cellular therapy have to be taken into account although the NRM observed in this study was low with 11%. Furthermore, this approach of allogeneic HSCT followed by ACI is vastly intricate and costly. Hence, further efforts have to be taken to improve prognosis of pediatric patients with relapsed or refractory solid tumors, such as ACI combined with antibodies e. g. dinutuximab or chimeric antigen receptor T cells directed against for example HER2/neu (ErbB2) expressed on some pediatric tumors [25] or CIK cells [22, 26], both administered in the autologous setting.

## MATERIALS AND METHODS

### Patients

Allogeneic HSCTs were conducted at our transplant center between October 1, 2003 and January 1, 2014 after approval by the regulatory authorities (Paul-Ehrlich-Institute, Langen, Germany (EudraCT 2006-000393-76)), and the local ethics committee (no. 87/06). Subsequent ACIs were applied on a compassionate use basis, after written informed consent, and case-by-case evaluation by the regional board (Regierungspräsidium Darmstadt, Germany). Written informed consent for ACIs was given by the patient and/or the representative in law before starting the cell infusion.

### Chemotherapy conditioning

A reduced intensity conditioning regimen including fludarabine (5 × 30 mg/m^2^), thiotepa (2 × 5 mg/kg), and melphalan (2 × 70 mg/m^2^) was administered. From January 2014 on, the condition regimen in some patients included an additional three-day course of clofarabine (3 × 40 mg/m^2^), etoposide (3 × 100 mg/m^2^), and cyclophosphamide (3 × 400 mg/m^2^).

### Serotherapy

A monoclonal anti-CD3 antibody (Ab) (muromonab-CD3, Orthoclone OKT3^®^, Janssen-Cilag) was employed until December 2010. Since January 2011, ATG Fresenius (3 × 10 mg/kg) was administered instead of OKT-3^®^. From January 2014 on, the above-mentioned chemotherapy regimens were followed by a five-day course with monoclonal anti-CD52 Ab (alemtuzumab, Campath^®^, 5 × 0.1 mg/kg).

### Graft manipulation

The haploidentical graft was engineered by CD3/CD19-depletion using microbeads and the Clinimacs^®^ system (Miltenyi Biotec, Bergisch Gladbach, Gemany). Stem cell proportion in the graft was intended to be more than 7 × 10^6^ CD34^+^ cells per kg recipient body weight and the graft was supposed to contain less than 1 × 10^5^ CD3^+^ T cells per kg recipient body weight.

### Adoptive cellular immunotherapies and treatment stratification

DLI, NK and CIK cell infusions were generated as described before [27–29]. In brief, allogeneic NK cells were generated from unstimulated leukapheresis by a two-step purification procedure (CliniMACs, Miltenyi Biotec, Bergisch Gladbach, Germany) using immunomagnetic CD3 T cell depletion, followed by NK cell enrichment (CD56^+^) with or without *in vitro* IL-2 stimulation and expansion for 9–14 days [28, 29]. 50–100 mL blood of the respective stem cell donor was used for CIK cell generation: Peripheral blood mononuclear cells were isolated and activated by *in vitro* cytokine stimulation (INF-γ, anti-CD3, IL-2 and IL-15) an expanded over 10–12 days [27].

Due to the imminence risk for disease recurrence or progression, start of ACIs was recommended within 60 days after HSCT in consideration of the circumstances that patients were complete chimeras, and showed no or only mild signs of aGVHD not exceeding grade I aGVHD in parallel to leucocyte recovery. ACIs included DLI or NK cell infusions between October 1, 2003 and August 11, 2011. Since then, CIK cell infusions were given preferentially. The recommended starting dose of DLI was 1 × 10^6^ T cells/kg in cases of HLA-matched related donors, and 0.25 × 10^6^ T cells/kg in cases of HLA-haploidentical donors. The recommended starting dose of NK or CIK cell therapy was 10 × 10^6^ NK cells/kg or 5 × 10^6^ T cells/kg, respectively. Considering the risk for GVHD, the interval between respective ACI doses included a minimum of 3–4 weeks. A doubling of ACI doses was considered for subsequent infusion, if no additional signs of aGVHD had appeared.

### Response and disease-related follow-up analysis

FU analysis was performed according to the guidelines of the respective treatment protocols: Ultrasound (tumor site, regional lymph nodes, abdomen pelvis, initial metastases) or MRI/CT scan was performed every three to four months (alternating, if applicable) in the first year and every six months thereafter. Additional investigations (chest-X-ray/CT scan) were performed every six months if applicable.

### Monitoring of immune reconstitution

Flow cytometric analyses for immune monitoring were performed as previously described [30]. In brief, immune reconstitution monitoring of CD3^+^CD56^-^ T cells, CD3^+^CD8^+^ cytotoxic T cells, CD3^+^CD4^+^ helper T cells, CD3^-^CD56^+^ natural killer (NK) cells, CD3^+^CD56^+^ T-NK cells, CD19^+^ B cells, and CD14^+^ monocytes was performed for all patients. Furthermore, CD3^+^CD8^+^CD69^+^ early activated cytotoxic T (T8) cells, CD3^+^CD8^+^HLA-DR^+^ late activated T8 cells, CD3^+^CD8^+^CD45RA^+^CD62L^+^ naïve T8 cells, CD3^+^CD8^+^CD45RO^+^CD62L^-^ EM T8 cells, CD3^+^CD8^+^CD45RO^+^CD62L^+^ CM T8 cells, CD3^+^CD4^+^CD69^+^ early activated helper T (T4) cells, CD3^+^CD4^+^HLA-DR^+^ late activated T4 cells, CD3^+^CD4^+^CD45RA^+^CD62L^+^ naïve T4 cells, CD3^+^CD4^+^CD45RO^+^CD62L^-^ EM T4 cells, CD3^+^CD4^+^CD45RO^+^CD62L^+^ CM T4 cells, and CD3^+^CD4^+^CD25^+bright^CD127^dim/-^ regulatory T cells (Treg) were assessed 100 ± 50 and 200 ± 50 days after allogeneic HSCT. As childhood blood values strongly depend on age, each patient’s longitudinally determined measurement was calculated from its corresponded age-matched norm published by Huenecke *et al.* [31] to allow the comparison among cell counts from patients of different ages.

### Statistical analysis

PFS and OS was calculated from date of first infusion to date of disease progression or event, which for OS was relapse, death, or NRM, whichever occurred first, and for survival date of event was date of death from any cause. PFS and survival curves were estimated according to Kaplan–Meier and compared according to Mantel–Cox-Test (log rank-test). Cumulative incidence curves for relapse were estimated adjusting for competing risk of other events. *T*-tests were two-sided, with a 0.05 significance level. Analyses were carried out using Graph Pad Prism Version 6.

## SUPPLEMENTARY MATERIALS





## Abbreviations

ACI: adoptive cellular immunotherapy
OS: overall survival
PFS: progression-free survival
CR: complete remission
CIK: cytokine-induced killer
NK: natural killer
DLI: donor lymphocyte infusions
aGVHD: acute graft versus host disease
RMS: rhabdomyosarcoma
SS: synovial sarcoma
or ES: Ewing sarcoma
NB: neuroblastoma
HBL: hepatoblastoma
NPC: nasopharyngeal carcinoma
INRG: North American Intergroup Rhabdomyosarcoma Study Group
IRS: International Neuroblastoma Risk Group
INSS: International *Neuroblastoma Staging System*
CSF2: colony stimulating factor 2
IL: interleukin
IFN: interferon
HSCT: hematopoietic stem cell transplantation
GVT: graft verus tumor
VGPR: very good partial remission
PR: partial response
HLA: human leukocyte antigen
ATG: anti-thymocyte globulin
FU: follow up
CIR: cumulative incidence or relapse
MMF: mycophenolatemofetil
CSA: cyclosporine A
MSCs: mesenchymal stromal cells
ECP: extra corporal photopheresis
CMV: cytomegalovirus
NRM: non-relapse mortality
IR: immune reconstitution
CM: central memory
EM: effector memory
MHC: major histocompatibility complex
Ab: antibody
Treg: regulatory T cells

## References

[1] Malempati S, Hawkins DS Rhabdomyosarcoma: review of the Children’s Oncology Group (COG) Soft-Tissue Sarcoma Committee experience and rationale for current COG studies. Pediatr Blood Cancer. 2012; 59:5–10. 10.1002/pbc.24118. 22378628 PMC4008325

[2] Weigel BJ, Lyden E, Anderson JR, Meyer WH, Parham DM, Rodeberg DA, Michalski JM, Hawkins DS, Arndt CA Intensive Multiagent Therapy, Including Dose-Compressed Cycles of Ifosfamide/Etoposide and Vincristine/Doxorubicin/Cyclophosphamide, Irinotecan, and Radiation, in Patients With High-Risk Rhabdomyosarcoma: A Report From the Children’s Oncology Group. J Clin Oncol. 2016; 34:117–122. 10.1200/JCO.2015.63.4048. 26503200 PMC5070550

[3] Gaspar N, Hawkins DS, Dirksen U, Lewis IJ, Ferrari S, Le Deley MC, Kovar H, Grimer R, Whelan J, Claude L, Delattre O, Paulussen M, Picci P, et al Ewing sarcoma: current management and future approaches through collaboration. J Clin Oncol. 2015; 33:3036–46. 10.1200/JCO.2014.59.5256. 26304893

[4] Ladanyi M Fusions of the SYT and SSX genes in synovial sarcoma. Oncogene. 2001; 20:5755–5762. 10.1038/sj.onc.1204601. 11607825

[5] Ferrari A, De Salvo GL, Brennan B, van Noesel MM, De Paoli A, Casanova M, Francotte N, Kelsey A, Alaggio R, Oberlin O, Carli M, Ben-Arush M, Bergeron C, et al Synovial sarcoma in children and adolescents: the European Pediatric Soft Tissue Sarcoma Study Group prospective trial (EpSSG NRSTS 2005). Ann Oncol. 2015; 26:567–572. 10.1093/annonc/mdu562. 25488687

[6] Carli M, Colombatti R, Oberlin O, Bisogno G, Treuner J, Koscielniak E, Tridello G, Garaventa A, Pinkerton R, Stevens M European intergroup studies (MMT4-89 and MMT4-91) on childhood metastatic rhabdomyosarcoma: final results and analysis of prognostic factors. J Clin Oncol. 2004; 22:4787–4794. 10.1200/JCO.2004.04.083. 15570080

[7] Brennan B, Stevens M, Kelsey A, Stiller CA Synovial sarcoma in childhood and adolescence: a retrospective series of 77 patients registered by the Children’s Cancer and Leukaemia Group between 1991 and 2006. Pediatr Blood Cancer. 2010; 55:85–90. 10.1002/pbc.22453. 20213848

[8] Cohn SL, Pearson AD, London WB, Monclair T, Ambros PF, Brodeur GM, Faldum A, Hero B, Iehara T, Machin D, Mosseri V, Simon T, Garaventa A, et al, and INRG Task Force The International Neuroblastoma Risk Group (INRG) classification system: an INRG Task Force report. J Clin Oncol. 2009; 27:289–97. 10.1200/JCO.2008.16.6785. 19047291 PMC2650388

[9] Yu AL, Gilman AL, Ozkaynak MF, London WB, Kreissman SG, Chen HX, Smith M, Anderson B, Villablanca JG, Matthay KK, Shimada H, Grupp SA, Seeger R, et al, and Children’s Oncology Group Anti-GD2 antibody with GM-CSF, interleukin-2, and isotretinoin for neuroblastoma. N Engl J Med. 2010; 363:1324–34. 10.1056/NEJMoa0911123. 20879881 PMC3086629

[10] Hiyama E Pediatric hepatoblastoma: diagnosis and treatment. Transl Pediatr. 2014; 3:293–299. 10.3978/j.issn.2224-4336.2014.09.01. 26835349 PMC4728840

[11] Lang P, Pfeiffer M, Muller I, Schumm M, Ebinger M, Koscielniak E, Feuchtinger T, Foll J, Martin D, Handgretinger R Haploidentical stem cell transplantation in patients with pediatric solid tumors: preliminary results of a pilot study and analysis of graft versus tumor effects. Klin Padiatr. 2006; 218:321–326. 10.1055/s-2006-942256. 17080334

[12] Koscielniak E, Gross-Wieltsch U, Treuner J, Winkler P, Klingebiel T, Lang P, Bader P, Niethammer D, Handgretinger R Graft-versus-Ewing sarcoma effect and long-term remission induced by haploidentical stem-cell transplantation in a patient with relapse of metastatic disease. J Clin Oncol. 2005; 23:242–244. 10.1200/JCO.2005.05.940. 15625381

[13] Toporski J, Garkavij M, Tennvall J, Ora I, Gleisner KS, Dykes JH, Lenhoff S, Juliusson G, Scheding S, Turkiewicz D, Bekassy AN High-dose iodine-131-metaiodobenzylguanidine with haploidentical stem cell transplantation and posttransplant immunotherapy in children with relapsed/refractory neuroblastoma. Biol Blood Marrow Transplant. 2009; 15:1077–1085. 10.1016/j.bbmt.2009.05.007. 19660720

[14] Illhardt T, Toporski J, Feuchtinger T, Turkiewicz D, Teltschik HM, Ebinger M, Schwarze CP, Holzer U, Lode HN, Albert MH, Gruhn B, Urban C, Dykes JH, et al Haploidentical Stem Cell Transplantation for Refractory/Relapsed Neuroblastoma. Biol Blood Marrow Transplant. 2018; 24:1005–1012. 10.1016/j.bbmt.2017.12.805. 29307718

[15] Inaba H, Handgretinger R, Furman W, Hale G, Leung W Allogeneic graft-versus-hepatoblastoma effect. Pediatr Blood Cancer. 2006; 46:501–505. 10.1002/pbc.20404. 15806543

[16] Cheng M, Chen Y, Xiao W, Sun R, Tian Z NK cell-based immunotherapy for malignant diseases. Cell Mol Immunol. 2013; 10:230–252. 10.1038/cmi.2013.10. 23604045 PMC4076738

[17] Schmidt-Wolf IG, Negrin RS, Kiem HP, Blume KG, Weissman IL Use of a SCID mouse/human lymphoma model to evaluate cytokine-induced killer cells with potent antitumor cell activity. J Exp Med. 1991; 174:139–149. 10.1084/jem.174.1.139. 1711560 PMC2118875

[18] Dierckx de Casterlé I, Billiau AD, Sprangers B Recipient and donor cells in the graft-versus-solid tumor effect: it takes two to tango. Blood Rev. 2018; 32:449–56. 10.1016/j.blre.2018.04.002. 29678553

[19] Merker M, Meister MT, Rettinger E, Jarisch A, Soerensen J, Willasch A, Huenecke S, Cappel C, Bremm M, Salzmann-Manrique E, Krenn T, Rossig C, Kremens B, et al Haploidentical allogeneic hematopoietic stem cell transplantation in patients with high-risk soft tissue sarcomas: results of a single-center prospective trial. Bone Marrow Transplant. 2018; 53:891–894. 10.1038/s41409-018-0088-6. 29367709

[20] Kloess S, Huenecke S, Piechulek D, Esser R, Koch J, Brehm C, Soerensen J, Gardlowski T, Brinkmann A, Bader P, Passweg J, Klingebiel T, Schwabe D, Koehl U IL-2-activated haploidentical NK cells restore NKG2D-mediated NK-cell cytotoxicity in neuroblastoma patients by scavenging of plasma MICA. Eur J Immunol. 2010; 40:3255–3267. 10.1002/eji.201040568. 21061445

[21] Brehm C, Huenecke S, Quaiser A, Esser R, Bremm M, Kloess S, Soerensen J, Kreyenberg H, Seidl C, Becker PS, Muhl H, Klingebiel T, Bader P, et al IL-2 stimulated but not unstimulated NK cells induce selective disappearance of peripheral blood cells: concomitant results to a phase I/II study. PLoS One. 2011; 6:e27351. 10.1371/journal.pone.0027351. 22096557 PMC3212563

[22] Kuci S, Rettinger E, Voss B, Weber G, Stais M, Kreyenberg H, Willasch A, Kuci Z, Koscielniak E, Kloss S, von Laer D, Klingebiel T, Bader P Efficient lysis of rhabdomyosarcoma cells by cytokine-induced killer cells: implications for adoptive immunotherapy after allogeneic stem cell transplantation. Haematologica. 2010; 95:1579–1586. 10.3324/haematol.2009.019885. 20378565 PMC2930961

[23] Schober SJ, von Luettichau I, Wawer A, Steinhauser M, Salat C, Schwinger W, Ussowicz M, Antunovic P, Castagna L, Kolb HJ, Burdach SEG, Thiel U Donor lymphocyte infusions in adolescents and young adults for control of advanced pediatric sarcoma. Oncotarget. 2018; 9:22741–22748. 10.18632/oncotarget.25228. 29854312 PMC5978262

[24] Klingebiel T, Boos J, Beske F, Hallmen E, Int-Veen C, Dantonello T, Treuner J, Gadner H, Marky I, Kazanowska B, Koscielniak E Treatment of children with metastatic soft tissue sarcoma with oral maintenance compared to high dose chemotherapy: report of the HD CWS-96 trial. Pediatr Blood Cancer. 2008; 50:739–745. 10.1002/pbc.21494. 18286501

[25] Ahmed N, Brawley VS, Hegde M, Robertson C, Ghazi A, Gerken C, Liu E, Dakhova O, Ashoori A, Corder A, Gray T, Wu MF, Liu H, et al Human Epidermal Growth Factor Receptor 2 (HER2) -Specific Chimeric Antigen Receptor-Modified T Cells for the Immunotherapy of HER2-Positive Sarcoma. J Clin Oncol. 2015; 33:1688–1696. 10.1200/JCO.2014.58.0225. 25800760 PMC4429176

[26] Rettinger E, Meyer V, Kreyenberg H, Volk A, Kuci S, Willasch A, Koscielniak E, Fulda S, Wels WS, Boenig H, Klingebiel T, Bader P Cytotoxic Capacity of IL-15-Stimulated Cytokine-Induced Killer Cells Against Human Acute Myeloid Leukemia and Rhabdomyosarcoma in Humanized Preclinical Mouse Models. Front Oncol. 2012; 2:32. 10.3389/fonc.2012.00032. 22655268 PMC3356002

[27] Rettinger E, Bonig H, Wehner S, Lucchini G, Willasch A, Jarisch A, Soerensen J, Esser R, Rossig C, Klingebiel T, Bader P Feasibility of IL-15-activated cytokine-induced killer cell infusions after haploidentical stem cell transplantation. Bone Marrow Transplant. 2013; 48:1141–1143. 10.1038/bmt.2013.19. 23474803

[28] Koehl U, Esser R, Zimmermann S, Tonn T, Kotchetkov R, Bartling T, Sorensen J, Gruttner HP, Bader P, Seifried E, Martin H, Lang P, Passweg JR, et al *Ex vivo* expansion of highly purified NK cells for immunotherapy after haploidentical stem cell transplantation in children . Klin Padiatr. 2005; 217:345–350. 10.1055/s-2005-872520. 16307421

[29] Koehl U, Sorensen J, Esser R, Zimmermann S, Gruttner HP, Tonn T, Seidl C, Seifried E, Klingebiel T, Schwabe D IL-2 activated NK cell immunotherapy of three children after haploidentical stem cell transplantation. Blood Cells Mol Dis. 2004; 33:261–266. 10.1016/j.bcmd.2004.08.013. 15528141

[30] Koenig M, Huenecke S, Salzmann-Manrique E, Esser R, Quaritsch R, Steinhilber D, Radeke HH, Martin H, Bader P, Klingebiel T, Schwabe D, Schneider G, Lehrnbecher T, et al Multivariate analyses of immune reconstitution in children after allo-SCT: risk-estimation based on age-matched leukocyte sub-populations. Bone Marrow Transplant. 2010; 45:613–621. 10.1038/bmt.2009.204. 19701252

[31] Huenecke S, Behl M, Fadler C, Zimmermann SY, Bochennek K, Tramsen L, Esser R, Klarmann D, Kamper M, Sattler A, von Laer D, Klingebiel T, Lehrnbecher T, Koehl U Age-matched lymphocyte subpopulation reference values in childhood and adolescence: application of exponential regression analysis. Eur J Haematol. 2008; 80:532–539. 10.1111/j.1600-0609.2008.01052.x. 18284628

