# Supplementary material for: Adoptive cellular immunotherapy for refractory childhood cancers: a single center experience
Source: Oncotarget. 2019 Oct 22;10(58):6138–51. doi: 10.18632/oncotarget.27242 (PMC6817438; doi:10.18632/oncotarget.27242)
Supplement: Supplementary file 1 [file oncotarget-10-6138-s001.pdf]

Adoptive cellular immunotherapy for refractory childhood cancers: a single center experience

SUPPLEMENTARY MATERIALS

Supplementary Table 1: Patients outcome, *n* = 18. See Supplementary Table 1
